# Mucous membrane pemphigoid in a patient with chronic hepatitis B virus infection: A case report

**DOI:** 10.1097/MD.0000000000025955

**Published:** 2021-05-21

**Authors:** Christine Yi-Ting Chou, Chi-Wei Lin, Gwo-Shing Chen, Ru-Yi Huang

**Affiliations:** a Department of Family and Community Medicine, E-Da Hospital; b School of Medicine, College of Medicine, I-Shou Univeristy; c Department of Dermatology, Kaohsiung Medical University Hospital; d Department of Dermatology, E-Da Hospital, Kaohsiung, Taiwan.

**Keywords:** mucous membrane pemphigoid, cicatricial pemphigoid, hepatitis B virus

## Abstract

**Rationale:**

Mucous membrane pemphigoid (MMP) is a rare, autoimmune bullous disease that affects mucosal surfaces and skin. Early and aggressive treatment initiation may be warranted due to the risks of serious complications. However, it can be challenging to make an initial diagnosis. Viral infection such as hepatitis B virus (HBV) infection has been found to be associated with the formation of autoimmune bullous diseases.

**Patient concerns:**

The patient was a 43-year-old male with gingivitis and recurrent swelling over the neck, cheeks, lips, and eyelids. The patient presented at oral medicine, otolaryngology, plastic surgery, and ophthalmology sequentially, and was later referred to the rheumatology, dermatology, and family medicine departments. Recurrent hemorrhagic bullae on oral mucosa and skin scarring occurred 2 years after the onset of the initial symptoms.

**Diagnosis:**

Skin biopsy with direct immunofluorescence was performed under the suspicion of MMP. Lesional hematoxylin and eosin stain and perilesional direct immunofluorescence were consistent with MMP.

**Interventions:**

Systemic Prednisolone and topical corticosteroid were used to control the disease.

**Outcomes:**

A flare-up of hepatitis B developed as a result of systemic prednisolone use. The disease went through relapses and remissions. The patient is on low-dose prednisolone (5 mg/day) with a monthly outpatient visit in the family medicine department.

**Lessons:**

It would be useful for medical practitioners in different specialties to be alert of the heterogeneous presentations of MMP. Chronic HBV infection might be a risk factor for MMP. In patients with chronic HBV infection, treatment of MMP must be closely monitored for the risk of reactivation of HBV.

## 1 Introduction

Mucous membrane pemphigoid (MMP) is a rare, autoimmune disease that affects mucosal surfaces, with occasional skin involvement. The disease may produce scarring, which in some circumstances can cause significant disability or even death. Early and aggressive treatment initiation may be warranted due to the risks of serious complications, such as blindness and airway compromise.^[1,2]^ However, it can be challenging to make an initial diagnosis. Moreover, the treatment regarding systemic immunosuppressants may need to be assessed thoroughly with the specific underlying chronic condition.^[3]^ Here we present a case with recurrent swellings of the neck, cheeks, eyelids, and lips, followed by the formation of hemorrhagic bullae with scar over oral mucosa and skin. The patient was diagnosed with mucous membrane pemphigoid 2 years after the onset of the initial symptoms. The treatment course was complicated with a progressive hepatitis B under concurrent immunosuppression.

## 2 Case

A 43-year-old Asian male presented at the outpatient department of dental medicine with recurrent painful swelling over the left cheek for six months. He denied fever, travel, contact, or recent trauma history. The patient is a chronic hepatitis B carrier. He works as an aircraft maintenance personnel. On examination, fistula over buccal aspect of tooth 37 (FDI World Dental Federation notation) with scant purulent discharge was noted. One firm mass over left buccal mucosa in the size of 1.5 x 1.5 cm was palpated. Amoxicillin/clavulanic acid, naproxen, and prednisolone were given for a total of 5 weeks. The mass failed to resolve; thus, the oral surgeon consulted the plastic surgeon for debridement. The plastic surgeon performed the excisional biopsy, which revealed minor salivary gland tissue and soft tissue with chronic inflammatory cell infiltration.

**Figure 1:**
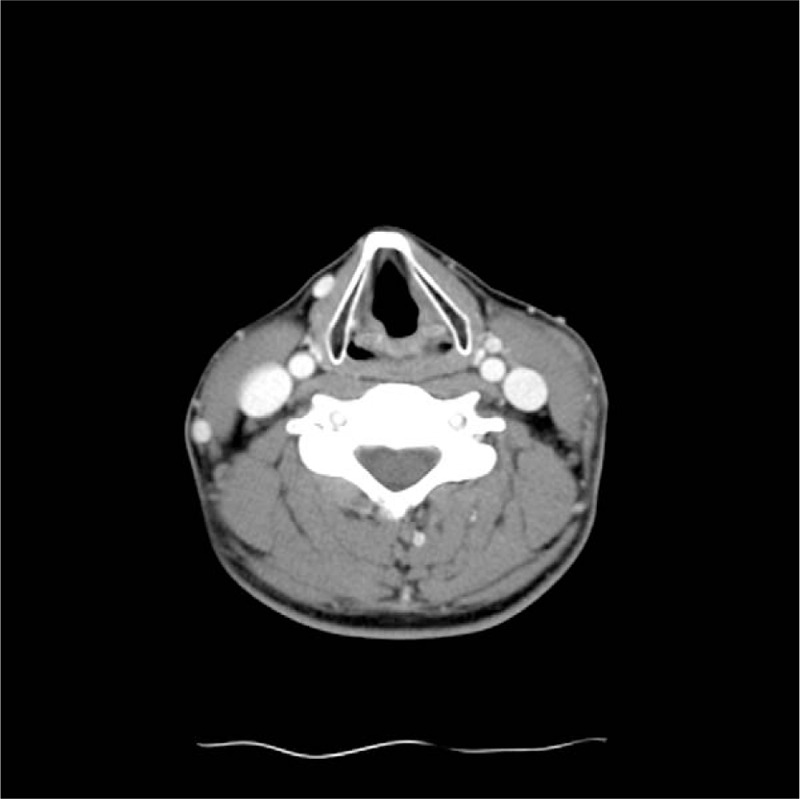
Computed tomography of head and neck shows slightly asymmetric thickening of left platysma muscle with overlying subcutaneous infiltrations containing a branch of external jugular vein.

Four weeks later, the patient visited the Otolaryngology Department, with puffy swelling of the right lower eyelid and a firm mass, 2.5 cm in diameter, at the left neck. The ear-nose-throat field was grossly normal, and the nasopharyngoscopy found no abnormality. Serum markers including antinuclear antibody, rheumatoid factor, anti-SSA antibody, anti-SSB antibody, and erythrocyte sedimentation rate were normal. The computerized tomography scan was ordered and showed left lateral neck focal inflammation (Fig. 1). Under the impression of inflammatory pseudotumor, the otolaryngologist prescribed prednisolone 30 mg/day (0.5 mg/kg/day) for 8 weeks and then gradually tapered the prednisolone dose. During the treatment course, the patient experienced hand cramps and rash on the back, which were considered as possible side effects of systemic steroids. The patient also experienced reddish swelling of the left eyelids and mild sore throat. He also visited the ophthalmology department for dryness and irritation of the eyes.

After prednisolone was tapered, however, the patient had a relapse of swelling right upper cheek, left lower cheek, and upper lip. The patient was referred to the rheumatologist and the dose of Prednisolone was escalated to 45 mg/day (0.75 mg/kg/day). The serum tests for cytoplasmic antineutrophil cytoplasmic antibodies (cANCA) and perinuclear antineutrophil cytoplasmic antibodies (pANCA) were both negative. Cryoglobulin was positive but turned negative on a second test. Four weeks later, alanine aminotransferase (ALT) was found elevated to 71 U/L, compared to 32 U/L 1 month ago. Although the Prednisolone was slowly titrated at the rate of 10 mg every 4 weeks, ALT continued to rise.

The rheumatologist then consulted the gastroenterologist and continued titration of prednisolone at a rate of 10 mg every 4 weeks. Laboratory data were obtained by the gastroenterologist and showed ALT 354 U/L, aspartate aminotransferase 116 U/L, total bilirubin 1.71 mg/dL, hepatitis B virus (HBV) viral load 18,900,000 IU/mL, HBsAg positive, Anti-HBe positive, HBeAg negative, Anti- hepatitis C virus (HCV) negative. The progressive hepatitis is suspected to be steroid-related. The gastroenterologist prescribed entecavir 0.5 mg/day and ursodeoxycholic acid 600 mg/day. Due to the progression of the patient's hepatitis B, the rheumatologist discontinued the prednisolone. Three weeks after, the aminotransferases returned to normal (ALT 34 U/L, aspartate aminotransferase 28 U/L). During the treatment of HBV, the patient experienced severe off-and-on swellings over eyelids and lower lip, as well as bullous skin lesions. However, the history of progressive hepatitis B limits the use of systemic immunosuppressants.

**Figure 2:**
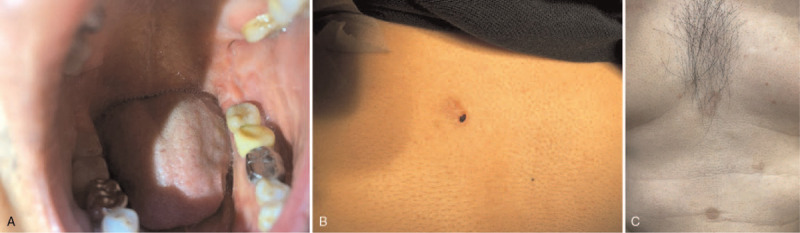
(A) Oral ulcers. (B) Bullae scar on the upper back, which was biopsied for histology and DIF. (C) Scarring bullae on the chest and abdomen.

**Figure 3:**
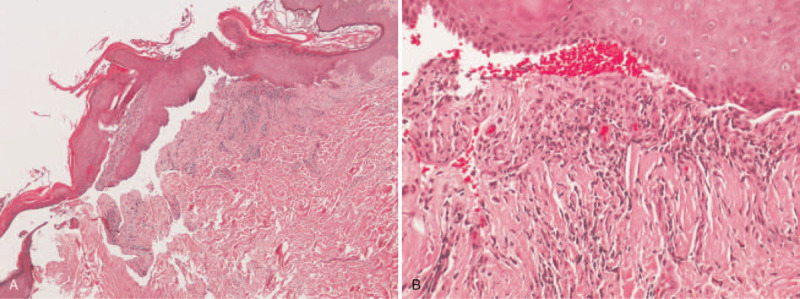
(A) Bullae with the detachment of the epidermis from the dermis. The dermis shows fibrosis. (B) Inflammatory cells are seen in the epidermal-dermal junction.

After the symptoms stabilized, the patient visited the family medicine department and mentioned recurrent hemorrhagic bullae on the oral mucosa. Upon examination, oral ulcers were seen, for which the family physician prescribed topical corticosteroid (Fig. 2A). The dermatologist was consulted after a visual report of bullae with a superficial scar formation on the trunk. The dermatologist performed a skin biopsy on the upper back with direct immunofluorescence (DIF) under the suspicion of MMP (Fig. 2B and C). The lesional specimen with hematoxylin and eosin stain showed bullae with the detachment of the epidermis from the dermis. Mild inflammation was seen (Fig. 3A and B). DIF of the perilesional biopsy shows immunoglobulin A: negative; immunoglobulin G: linear deposition at the dermal-epidermal junction; immunoglobulin M: negative; C3: linear deposition at the dermal-epidermal junction; fibrinogen: (−). The result was consistent with MMP.

On the diagnosis of MMP, the patient has received a tapered dosage of oral prednisolone (5 mg/day) at the family medicine department. He is under a relatively stable condition with no active new lesions other than infrequent self-limiting oral ulcers. The ophthalmologist examined and found no signs of ocular involvement of MMP. Given the potential adverse sequelae of MMP, including ocular, esophageal or urogenital erosions and compromised airway, the patient is advised to take monthly outpatient visits and watch out for any signs of active disease, including ocular, oral, and extraoral involvement.

## 3 Discussion

The patient presented to the dental department with gingivitis and recurrent swelling of the cheek, followed by the otolaryngology department with masses over the cheek and the neck. He then received the combined care between the rheumatologist and the gastroenterologist because of the prolonged symptoms and progressive hepatitis. Two years later, on the notion of the bullae, the family physician referred him to the dermatologist. The diagnosis of MMP was made 2 years after the onset of the initial symptoms. This course highlights the challenge it posts to direct the initially nonspecific symptoms to the final diagnosis of MMP, a disease that can lead to severe functional impairment.

Mucous membrane pemphigoid (MMP) is a subepithelial blistering and erosive disorder that affects the mucosal surfaces and skin. The areas involved ranged from the gingiva, oral mucosa, tongue, palate, eyes, nose, nasopharynx, hypopharynx, larynx, esophagus, genitals, to the anus.^[4]^ The cutaneous involvement typically included the head, neck, or upper trunk. The disease is broadly heterogeneous regarding site and severity of involvement in patients. Historically, the term cicatricial pemphigoid was used to describe the disease due to the scarring sequelae that may develop.^[2]^

MMP generally affects middle-aged and elderly people. White people and females are more likely to have the disease.^[5–8]^ The estimated incidence rate in the general population is 2/million/year.^[5–8]^ Our patient's age, race, and sex do not fall in the most susceptible group. However, previous studies have suggested that virus infection such as HBV, HCV, *H pylori*, *T gondii*, and cytomegalovirus predisposes patients to the development of autoimmune bullous diseases, including bullous pemphigoid (BP) and pemphigus.^[9]^ In 2018, Jang et al^[10]^ described a patient with BP associated with chronic hepatitis C virus infection. Likewise, the carrier status of HBV of our patient is likely one of the risk factors associated with the formation of MMP.

The onset of MMP is gradual, followed by acute exacerbations and remissions. In the majority of patients, the oral mucosa is the site of onset and is the most frequently involved (85%) site.^[4,11]^ As in our case, detecting intact vesicles or bullae on the mucosa is uncommon.^[2]^ Gingival presentation is often desquamative, and in mild cases, manifests as erythema or edema.^[2]^ The patient presented to the oral medicine department with gingivitis, thus possibly the first sign seen in patients with MMP. Cutaneous involvement typically presents as tense bullae, which often heals with scarring. Common sites include the scalp, face, or upper trunk. In the absence of treatment, MMP is usually a chronic, progressive disease that results in functionally limiting or life-threatening disease.^[2,12]^

There is no diagnostic criteria for MMP.^[13]^ The international consensus is based upon the clinical, histological, and immunopathological findings. The essential components are a clinical picture of mucosa-dominated lesions and positive DIF for immunoglobulin G, immunoglobulin A, immunoglobulin M, or C3 at the basement membrane zone.^[1,13]^ Both a lesional tissue specimen for hematoxylin and eosin staining and a perilesional tissue specimen for DIF examinations should be obtained. Other than the classical diagnosis, some experts suggest serum testing, including indirect immunofluorescence and enzyme-linked immunosorbent assay, to provide additional information. Indirect immunofluorescence helps identify patients at risk for malignancy-associated laminin 332 (epiligrin) MMP.^[13]^

There is limited evidence to guide therapy in MMP.^[14,15]^ Topical corticosteroids are the first-line local treatment for a mild disease with localized oral mucosa or skin involvement.^[1]^ Potential adverse effects of topical corticosteroids include mucosal atrophy, oropharyngeal candidiasis, and dyspepsia. Topical tacrolimus and intralesional corticosteroid are other options for patients who fail to respond adequately to topical corticosteroids. For moderate to severe disease which cannot be controlled with local therapy alone or with widespread oral disease, systemic glucocorticoids and dapsone are frequently used. For refractory disease, aggressive immunosuppressive regimens (ie, azathioprine, mycophenolate mofetil, and cyclophosphamide) and biologic therapies (ie, intravenous immune globulin and rituximab) can be considered. The potential serious adverse effects and high costs are the main concerns regarding the use of these agents.^[16]^ In our case, the chronic infection of HBV causes a treatment challenge since immunosuppressants can induce the reactivation of virus infections such as HBV and HCV.^[5]^

In our case, the initial presentation of the disease is nonspecific to MMP. It suggests that chronic inflammation might be the only early sign in MMP, if no blisters or erosions could be seen in the clinical settings. As the literature describes, the disease course is fluctuating with relapses and remissions.^[4]^ The broadly heterogeneous involvement and severity of MMP is the main challenge for medical practitioners to make a tentative diagnosis. Moreover, the patient presenting to different specialties (oral medicine, otolaryngology, rheumatology, ophthalmology, dermatology, and family medicine) makes this case noteworthy. It urges medical practitioners in various specialties to understand this disease, hence making early diagnosis and treatment possible. It is also notable that the patient is a chronic HBV carrier, which poses a risk of reactivation of HBV during systemic therapy. Therefore, it further highlights the need for multidisciplinary care of patients with MMP.

## 4 Conclusion

MMP is a rare autoimmune disease which affects the mucosal surfaces and occasionally the skin. It typically occurs in middle-aged or elderly patients. Virus infections, such as HBV and HCV infection, may contribute to the formation of MMP. The disease is widely heterogeneous and thus can cause a challenge for diagnosis. Clinical practitioners in all specialties should be aware of the disease's characters since early and aggressive treatment is vital to prevent functionally impairing complications such as blindness and compromised airway. For patients with chronic HBV infection and MMP, subspecialists need to closely monitor the infection's possible reactivation. Multidisciplinary care is crucial in the management of MMP.

## Acknowledgments

The authors thank Dr. Tsung-Heng Tsai for providing the pathology photos and Tara Chen for grammar suggestions.

## Author contributions

**Conceptualization:** Christine Yi-Ting Chou.

**Investigation:** Christine Yi-Ting Chou.

**Supervision:** Chi-Wei Lin.

**Validation:** Gwo-Shing Chen.

**Writing – original draft:** Christine Yi-Ting Chou.

**Writing – review & editing:** Christine Yi-Ting Chou, RuYi Huang.
